# Experimental Study on the Mix Ratio of Restored Heritage Building Adobe

**DOI:** 10.3390/ma15114034

**Published:** 2022-06-06

**Authors:** Jianwei Yue, Yiang Zhang, Peng Li, Jing Zhang, Xuanjia Huang, Yang Yue, Zhiguang Han

**Affiliations:** 1 School of Civil Engineering and Architecture, Henan University, Kaifeng 475004, China; yjw@vip.henu.edu.cn (J.Y.); zya@henu.edu.cn (Y.Z.); ZJING864@126.com (J.Z.); hxj_henu@126.com (X.H.); hanzhiguang01@163.com (Z.H.); 2 Key Laboratory for Restoration and Safety Evaluation of Immovable Cultural Relics in Kaifeng City, School of Civil Engineering and Architecture, Henan University, Kaifeng 475004, China; 3 Key Research Institute of Yellow River Civilization and Sustainable Development, School of Civil Engineering and Architecture, Henan University, Kaifeng 475004, China; 4 Department of Mechanical Engineering, University College London, Torrington Place, London WC1E 7JE, UK; yjwchn@126.com

**Keywords:** unconfined compressive strength, water resistance, mix ratio, modified raw adobe

## Abstract

The reciprocating action of the external environment gradually reduces the mechanical properties and water stability of original heritage buildings, resulting in the gradual loss of their cultural value. In this paper, the adobe for the construction of raw soil and cultural relics in western Henan is taken as the research object. The local plain soil is used as the raw material, and the adobe samples are prepared with modified materials such as quicklime and sodium methyl silicate, in order to improve its mechanical properties and water stability. The degree of correlation between the compressive strength, capillary water absorption, pH value, particle size distribution, and the electrical conductivity of modified raw adobe, as well as the modification mechanism of the microstructure, was studied. The results show that the addition of quicklime and sodium methyl silicate can enhance the compressive strength and water resistance of the modified raw adobe, and the optimum dosage is 1.5% sodium methyl silicate; with the increase of the curing age, the compressive strength of the single-mixed quicklime sample, the single mixed sodium methyl silicate samples, and the composite sample were increased by 1.94 times, 12.6 times and 2.61 times, respectively, compared with the plain soil samples, and with the increase of compressive strength, the pH, conductivity and capillary water absorption of the samples decreased continuously. It is evident from the particle gradation test and SEM images that the internal pores of the samples in the modified group become smaller, and the particle structure of the sample doped with sodium methyl silicate is the densest. The results of the study provide support for the restoration of the soil and cultural-relic buildings.

## 1. Introduction

**Figure 1:**
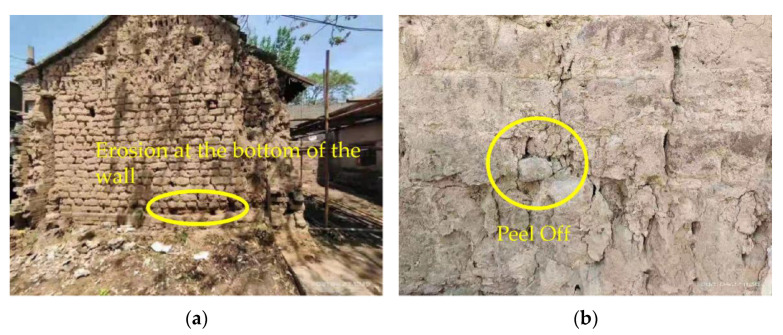
Disease map of cultural-relic buildings in western Henan: (**a**) erosion at the bottom of the wall; (**b**) peeling off the wall.

Raw soil is one of the earliest building materials used by human beings, and it is widely used because of its characteristic thermal insulation, wide range of materials, raw environmental protection, and recyclability [1]. Many soil- composed cultural relic buildings that have survived to this day are also raw soil buildings. There are many soil-composed and cultural relic buildings in Henan Province, such as the Qingtai Site in Xingyang City and the Shizhuang Site in Zhoukou City. These raw soil sites are very vulnerable to the erosion and damage of rainwater, especially in the face of the “7.20” heavy rainstorm disaster. These raw soil sites have been seriously threatened and damaged to varying degrees. Compared with soil sites in the arid regions of northwestern China, soil heritage buildings in Henan Province are more susceptible to climate and groundwater impacts, resulting in the destruction of the walls of soil heritage buildings (Figure 1). There are two main reasons: on the one hand, Henan Province belongs to a typical temperate monsoon climate, with cold and dry winters, hot and rainy summers, and more concentrated rainfall throughout the year. Meteorological disasters such as heavy rains are common [2]; on the other hand, the abundant underground capillary water carries soil soluble salts through capillary action, so that it is enriched at the bottom of the raw soil wall. With the change in temperature and moisture, the cyclic salt-action causes the bottom surface of the raw soil wall to become wet and fall off. This results in damage to the soil and cultural-relic buildings. It is evident that water-damage to the soil and cultural-relic buildings is the most obvious and unavoidable. Therefore, the question of how to repair the raw soil heritagel buildings and improve the strength of the raw adobe [3,4] and its water resistance [5,6,7] has become an engineering problem that urgently needs to be solved.

In recent years, scholars at home and abroad have used different materials to repair and reinforce the damaged soil and cultural-relic buildings. Chen et al. [8] have reported that mixing the SH solution with the site soil can improve the mechanical properties and water stability of the site soil, but its compatibility with the soil site needs to be considered when reinforcing the site. Liu et al. [9] improved the water resistance of the San he soil site by using microbial-induced calcium carbonate precipitation technology, but the soil samples after bacterial improvement had a strong odor, and the improved strength was limited. Niu et al. [10] improved the disintegration characteristics of soil sites in Northwestern China with a glutinous rice slurry, which provided a theoretical basis for the application of glutinous rice solution in the protection of soil sites. Kong et al. [11] improved the shear strength and compressive strength of the site soil by adding lime and starch ether to the silt. Qian et al. [12] conducted a study on the use of desulfurization waste to modify raw soil materials, and the results showed that the calcined desulfurization gypsum and the content of solid sulfur significantly improved the compressive strength of raw soil materials. Zhou et al. [13] determined the optimal soil ratio by studying the suitable mortar ratio for machine-made adobe and improved the compressive strength and shear strength of the raw soil structure. Yang et al. [14] determined that the compressive strength of modified raw adobe with a curing age of 28 d and 20% lime alone was the best by studying the influence of lime-based materials on the mechanical properties of modified raw adobe.

**Figure 2:**
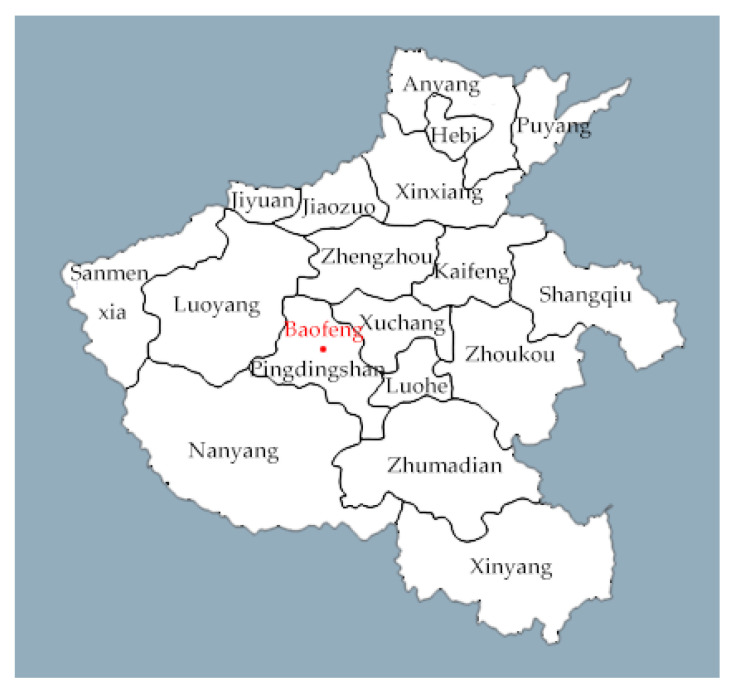
Location map of the study area.

In summation, in the field of soil heritage protection, there are few studies considering the strength and water resistance of adobe. Therefore, the focus of this paper is to ensure the strength of raw adobe while improving its water resistance. In this paper, taking the raw soil cultural-relic buildings in Baofeng County, Pingdingshan City, and Henan Province (Figure 2) as the research objects, an imitation site-soil was prepared. In addition, the traditional wet method was used to make the clay, adding quicklime, sodium methyl silicate, and wheat straw to the clay to explore the changes in its mechanical properties and water resistance. A compression test and a capillary water absorption test were carried out, and a good mix ratio was obtained, which provided a reference for the restoration of the cultural heritage buildings in western Henan.

## 2. Materials and Methods

### 2.1. Experiment Material

#### 2.1.1. Soil

**Figure 3:**
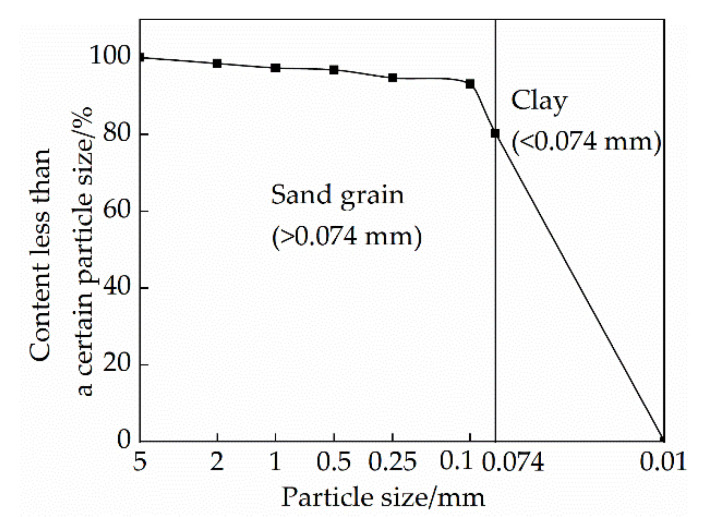
Particle size distribution of soil.

The soil used in the experiment was taken from the rural soil near the site of raw soil and cultural relics in western Henan, and the soil depth was below 20 cm–30 cm to ensure that the various physical properties of the undisturbed soil were from the same type of soil. Geotechnical tests were carried out in accordance with the “Standards for Geotechnical Test Methods” (GB/T50123-2019), and the physical and mechanical properties of the soil samples are shown in Table 1. The particle gradation curve of soil is shown in Figure 3.

#### 2.1.2. Quicklime

The addition of quicklime can cause a series of chemical reactions in the sample to generate calcium carbonate crystals as an expansive product, thereby improving the mechanical properties of the modified green adobe. Its chemical reaction equation is as follows:

(1) $$\mathrm{CaO}\;+{\;H}_{2}O\;=\;\mathrm{Ca}{\left(\mathrm{OH}\right)}_{2}$$

(2) $$\mathrm{Ca}{\left(\mathrm{OH}\right)}_{2}+{\;\mathrm{CO}}_{2}={\;\mathrm{CaCO}}_{3}↓+{\;H}_{2}O$$

The main physical and chemical indicators are as follows: the relative molecular mass is 56.08, the CaO content is not less than 98%, and the ignition loss is 2%.

#### 2.1.3. Sodium Methyl Silicate

Sodium methyl silicate is a new type of rigid building waterproof material with good penetrating crystallinity. The silanol group in its molecular structure reacts with the silanol group in the silicate material through dehydration and crosslinking, thereby forming an excellent hydrophobic layer, which has the functions of micro-swelling and increasing compactness [15,16]. Its chemical reaction equation is as follows:

(3) $$2{\mathrm{CH}}_{3}\mathrm{Si}{\left(\mathrm{OH}\right)}_{2}\mathrm{ONa}\;+{\;\mathrm{CO}}_{2}+{\;H}_{2}O\;\to \;2\left[{\mathrm{CH}}_{3}\mathrm{Si}{\left(\mathrm{OH}\right)}_{3}\right]+{\;\mathrm{Na}}_{2}{\mathrm{CO}}_{3}$$

(4) $$n\left[{\mathrm{CH}}_{3}\mathrm{Si}{\left(\mathrm{OH}\right)}_{3}\right]\to \left[{\mathrm{CH}}_{3}{\mathrm{SiO}}_{1.5}\right]n\;+1.5H_{2}O$$

The solid content (%) of sodium methyl silicate used in the sample is ≥30, and the pH value is 12~13.

#### 2.1.4. Straw

Through the previous statistical analysis of the traditional Chinese water de-drying production process, it was found that natural plant fibers such as grass tendon, wheat straw, wheat husk, and straw are often used as modified additives. These articles were purchased from a farm in Kaifeng City to ensure that the adobe production process was consistent with the traditional production process, technology, and materials. When making the bricks, the wheat straw was cut into sections, and each section was 2.5–3 cm.

### 2.2. Experiment Methods

#### 2.2.1. Sample Plan

To explore the effect of each admixture on the strength of the sample under different curing ages and different proportions, the macroscopic effect evaluation and microscopic mechanism exploration of modified raw adobe were carried out through an unconfined compressive strength test, pH value detection, sieving test, and electrical conductivity test. In this paper, quicklime, sodium methyl silicate, and wheat straw are used as admixtures. Among them, the content of quicklime is 7%, 15%, and 23%, the content of sodium methyl silicate is 0.5%, 1.5%, and 3%, and the content of wheat straw is the dosage is 0.5% [17]. The specific test plans and numbers are shown in Table 2.

#### 2.2.2. Sample Preparation

In this paper, the modified adobe was made by using the local traditional production process. The test steps of the three types of samples are as follows:

(1) When making the samples of the single-mixed quicklime group, mix 2 kg of the dried soil sample with a 5 mm sieve and quicklime evenly, add wheat straw and mix again. Then add plain water to control the moisture content of the prepared soil samples at 32% (the liquid limit of the soil). Considering that the reaction between quicklime and water consumes water and the reaction speed is fast, based on the reaction principle of CaO + H_2_O = Ca(OH)_2_, it has been concluded that 1 g of CaO will consume 0.32 g of water, so the water consumption of lime is also considered when adding water. When the temperature of the prepared soil samples drops to 25 °C, pour them into the mold, vibrate and compact them, demold immediately, and then carry out natural curing in the laboratory.
(2) When making the samples of the single-doped sodium methyl silicate group, first mix 2 kg of the dried soil sample with a 5 mm sieve and the wheat straw, and then dissolve the sodium methyl silicate solution in the added water and stir evenly. Finally, the mixed solution and the mixed soil sample are fully mixed before sample preparation.
(3) When making the sample of the compound blending group, first mix 2 kg of the dried soil sample with a 5 mm sieve and quicklime, and then fully mix it with the wheat straw, then dissolve the sodium methyl silicate in the added water and stir evenly. Finally, the mixed solution and the mixed soil sample are fully mixed, the sample is prepared, and the mold is released immediately. When the pure soil group was made, the moisture content was 32% of the dry soil, and the samples were prepared immediately.

#### 2.2.3. Unconfined Compressive Strength Test

The test method refers to GB/T 2542-2012, the Test Method for Wall Bricks, and the unrestricted compressive strength of the adobe is tested by an electro-hydraulic servo testing machine (MTS 810) with a loading rate of 0.5 mm/min. According to the test groups in Table 2, the unconfined compressive strengths at three different curing ages were measured, and there were three parallel samples at each age, totaling 84 pieces. In the test, two half-cuts of the same brick were stacked in opposite directions, and the stacked portion was not less than 100 mm.

#### 2.2.4. PH Test

Samples damaged after compression testing were sealed and stored for pH testing. 10 g of the damaged sample was added to 50 mL of deionized water for a mixture [18]. After standing, a Shanghai San Xin PH5S pen pH meter was used to test the pH value of the supernatant.

#### 2.2.5. Electrical Conductivity Test

10 g of damaged soil sample was put into a beaker, and deionized water was added according to the soil-water mass ratio of 1:5 and was fully stirred and then shaken for 3 min. Then, the conductivity of the supernatant was tested with the RS-485 soil moisture- temperature conductivity sensor of Shandong Jianda PeopleSoft Company [19], to explore the carbonation degree of the samples under different ratios and different curing ages.

#### 2.2.6. Particle Size Test

The particle gradation is strictly in accordance with the “Geotechnical Test Method Standard” (GB/T50123-2019) for screening experiments. 200 g of soil samples damaged after the unconfined compression test were rolled through a 5 mm sieve, shaken on a shock-type standard vibrating screen machine for 15 min, and then the cumulative sieve residue of each sieve was measured to explore the particle size distribution of modified adobe under different influencing factors. In addition, a fineness modulus is used to analyze the change of soil particles more accurately. The fineness modulus formula is as follows:

(5) $$MX_{n}=\frac{\left(A2+A3+A4+A5+A6-5A1\right)}{\left(100-A1\right)}$$

*MXn*: fineness modulus of soil particles.
*n:* curing age.
*A*: cumulative sieve residue of particles of a certain size.

#### 2.2.7. Capillary Water Absorption Test

To simulate the existing environment of the soil and cultural-relic buildings, the capillary water absorption test was carried out on the samples before and after improvement. The steps of the test process are as follows: place a row of permeable stones in a sink, add water to the sink, ensure that the water is flush with the permeable stones, and then put the non-woven fabric on the permeable stones. After drying the sample to measure its initial mass, place it on the non-woven fabric, and measure its mass every 0.5 h for 24 h [2]. Finally, the curve of the water absorption quality and water absorption time of different samples can be obtained.

#### 2.2.8. Microstructure Testing

The failure samples after the unconfined compressive strength test were subjected to an SEM scanning test to explore the evolution of the internal microstructure of the samples with different ratios.

## 3. Results and Analysis

### 3.1. Effects of Mix Ratio and Curing Age on the Strength of Modified Raw Adobe

**Figure 4:**
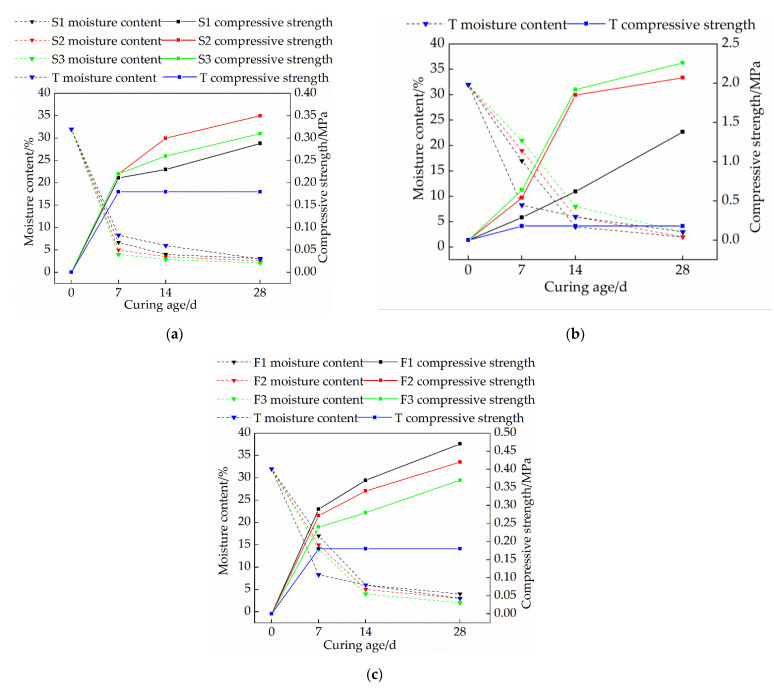
The relationship between the compressive strength and moisture content of each group and the curing age: (**a**) Single mixed quicklime group; (**b**) Mono-doped sodium methyl silicate group; (**c**) Compounding group.

**Figure 5:**
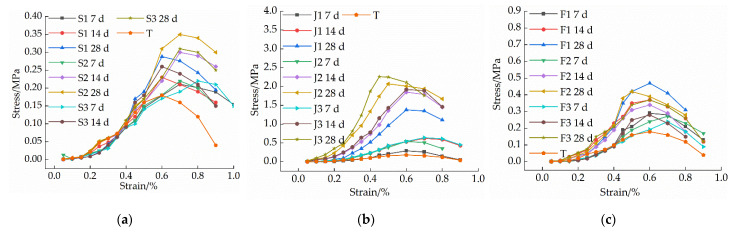
The stress-strain diagram of each group of samples: (**a**) Single mixed quicklime group; (**b**) Mono-doped sodium methyl silicate group; (**c**) Compounding group.

Figure 4a shows the relationship between the compressive strength and moisture content of the samples in the single-mixed quicklime group and the curing age, and the stress-strain relationship curve is shown in Figure 5a. The compressive strength of the plain soil group is not affected by the curing age and is 0.18 MPa. As it can be seen from Figure 4a and Figure 5a, the longer the curing time, the greater the peak stress of the stress-strain curve, and the earlier the peak point, and the more obvious the softening characteristics. Moreover, the compressive strength of the samples increased rapidly in the early stage, and the increased speed became slower after the curing age reached 14 d. The peak stress of the sample first increased and then decreased with the increase of the quicklime content. When the curing age is 28 d, the compressive strength of the S2 group is the highest, reaching 0.35 MPa, which is 1.94 times that of the plain soil group. The main reason for the above phenomenon is that the added quicklime generates CaCO_3_ after a series of hydration and carbonation reactions. CaCO_3_ crystals have a high strength and water stability, and have a cementation effect with soil particles, which greatly improves the integrity and strength of the soil [20,21,22]; however, when the amount of quicklime is higher than 15%, there will be a portion of the unreacted Ca(OH)_2_ in the lime soil, which will weaken the connection between soil particles. When this weakening effect exceeds the previous cementation effect of calcium carbonate it will lead to the reduction of the strength of the modified soil [23,24], and too much lime exists between the soil particles, which leads to the increase of the compressive deformation of the soil and the decrease of the unconfined compressive strength. In addition, the heat released during the chemical reaction of quicklime can cause micro-cracks inside the soil particles, which is also detrimental to the strength.

The relationship between the compressive strength and moisture content of the samples in the single-doped sodium methyl silicate group and the curing age is shown in Figure 4b, and the stress-strain relationship curve is shown in Figure 5b. As it can be seen from Figure 4b and Figure 5b the compressive strength of the sample increases with the increase of the sodium methyl silicate content; with the increase of the curing age, the peak stress of the soil sample is larger, and the peak point is advanced. When the curing age is 28 d, the peak stress of the J3 group is the highest, reaching 2.26 MPa, which is 12.6 times that of the plain soil group. This fully demonstrates that the addition of sodium methyl silicate can improve the compressive strength of the modified raw adobe. The analysis found that sodium methyl silicate can react with CO_2_ and water in the air to decompose into methyl silicic acid and quickly polymerize to form a polysiloxane (C5H12OSi) hydrophobic film, resulting in a certain degree of hydrophobicity [15]. In addition, sodium methyl silicate has good osmotic crystallinity and micro-expansion, which can block the formation of interconnected pores while reducing the internal pores of adobe and block the path of the water flow [25], thereby avoiding a certain amount of micro-cracks caused by excessive water loss, increasing the compactness of the adobe, and enhancing its strength. For single-doped sodium methyl silicate, with the increase of sodium methyl silicate content, its strength also increases continuously. The strength of the water repellent with a dosage of 1.5% and 3% is not very different, and both can reach about 2 MPa. For the purpose of cost-saving, for the compounding group, 1.5% water repellent was used to mix with lime.

The relationship between the compressive strength and moisture content of the compounded group after modification and the curing age is shown in Figure 4c, and the stress–strain relationship curve is shown in Figure 5c. As it can be seen from Figure 4c and Figure 5c, with the increase of quicklime content, the peak stress of the sample decreases; at the same dosage, the peak stress of the sample increases with the increase of the curing age. When the curing period is 28 d, the maximum compressive strength of the F1 group is 0.47 MPa, which is 1.62 times that of the 7 d period, 2.61 times that of the plain soil group, and 1.34 times that of the optimal group with single quicklime. The overall compressive strength of the compound-mixed group is higher than that of the single-mixed quicklime group, but it is far less than that of the single-mixed sodium methyl silicate group. The reason for this is that after adding quicklime, the reaction between quicklime and water consumes a lot of water, and a large amount of heat is released to evaporate part of the water, which causes the sample to lose water too quickly, resulting in micro-cracks inside the modified raw adobe and reducing the strength of the adobe. The addition of sodium methyl silicate can have a certain water retention effect for the adobe so that the water loss rate is slowed down, and the compressive strength of the sample can be improved after mixing with an appropriate amount of quicklime. However, with the increase of the amount of quicklime, the rate of water loss is accelerated, far exceeding the water retention effect of sodium methyl silicate, thereby reducing the strength.

### 3.2. Effects of Mix Ratio and Curing Age on pH Value of Modified Raw Adobe

**Figure 6:**
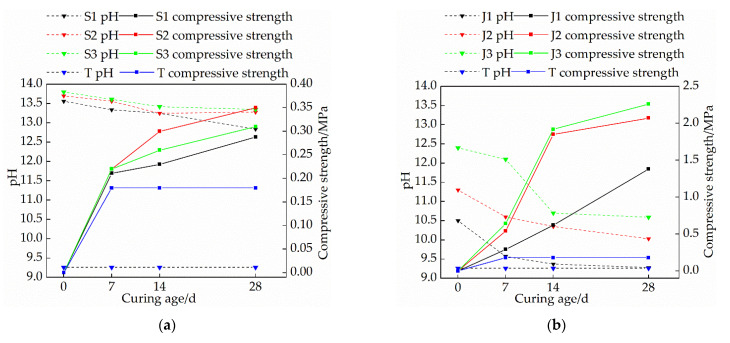

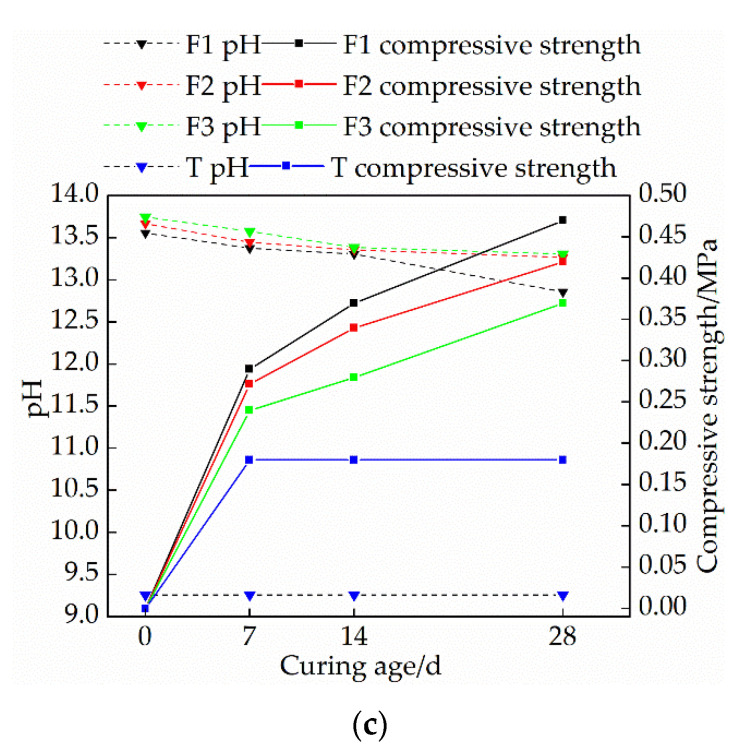
The relationship between compressive strength, pH, and curing age: (**a**) Single mixed quicklime group; (**b**) Mono-doped sodium methyl silicate group; (**c**) Compounding group.

Figure 6a is a graph showing the relationship between the compressive strength and pH of the single-mixed quicklime group and the curing age. As it can be seen from Figure 6a, the pH of the sample increases with the increase of the amount of quicklime; under the same dosage, the pH of the sample decreases with the increase of curing age; the compressive strength of the sample increases with the decrease of pH; thus, the greater the pH drop, the faster the compressive strength increase. From the change in the pH, it is evident that the pH value decreased rapidly from 0 to 14 days, and then the decreasing rate became subtle. This is because when the curing age is short, the water content in the sample is higher. After the CO_2_ gas in the air enters the sample, it quickly dissolves in water to form carbonic acid. The pH of the samples decreased rapidly. With the increase of the curing age, the moisture content inside the sample decreases, and the amount of carbonic acid formed by CO_2_ in the air dissolved in water is also relatively less, resulting in a limited amount of OH^−^ consumed, and thus the rate of the pH decline tends to be gentle. The faster the pH drop, the more calcium carbonate is produced, and the cohesion of calcium carbonate between soil particles makes the modified raw adobe stronger. In comparison, the pH increases with the increase of the content of quicklime because the reaction between quicklime and water is alkaline. However, it is not the case that an increasingly higher dosage leads to a correspondingly faster reaction rate. The addition of a small amount of quicklime has a great influence on the mechanical properties of the modified raw adobe [14]. If the amount of quicklime is too high, too much water will be consumed when OH^−^ is generated, and due to the rapid water loss, the amount of carbonic acid formed with CO_2_ in the air is relatively limited, and the amount of calcium carbonate provided is relatively small. Therefore, the pH value decreases slowly.

Figure 6b is a graph showing the relationship between the compressive strength, the pH of the sodium methyl silicate single-doped group, and the curing age. Combined with the change curve of the moisture content in Figure 4b, it is evident that the pH value of the sample increases with the increase of the sodium methyl silicate content; under the same dosage, the pH value of the sample decreased with the increase of the curing age, and the pH value of the sample decreased rapidly within 14 days, and the decrease rate became gentle after 14 days; the compressive strength of the samples increased with a decreasing pH. Sodium methyl silicate can chemically react with CO_2_ in water and air to generate polymethylsilyl (C5H12OSi) ether inside the adobe, which can increase the compactness between soil particles. The formation of polymethylsilyl (C5H12OSi) ether lowers the pH of the sample, so as the pH decreases, the more polymethylsilyl (C5H12OSi) ether produced, and the higher the compressive strength of the sample.

Figure 6c is a graph showing the relationship between the compressive strength and pH of the compounding group and the curing age. Combined with the change of water content in Figure 4c, it is evident that the pH of each compounding group increases with the increase of the amount of quicklime; under the same dosage, the pH of the sample decreases with the increase of the curing age; and the compressive strength of the samples increased with a decreasing pH. The pH of the samples in the F2 and F3 groups decreased rapidly within 14 days and then slowed after 14 days, while the pH value of the samples in the F1 group increased rapidly after 14 days. This is because the reaction of quicklime and sodium methyl silicate in the adobe will consume water. When the amount of quicklime increases, the water in the sample will be quickly lost, resulting in the inability for the next reaction to occur [26]; therefore, the pH decreases slowly. After 7% quicklime was added, there was still more water in the sample at 14 d, which provided the conditions for the next carbonization reaction. Therefore, for the composite sample, the lower the initial pH of the sample, the faster the pH decline with the increase of the curing age, and the higher the strength of the modified raw body.

### 3.3. Effects of Mix Ratio and Age on Particle Gradation of Modified Raw Adobe

#### 3.3.1. Influence of Lime on Particle Size Distribution of Samples

**Figure 7:**
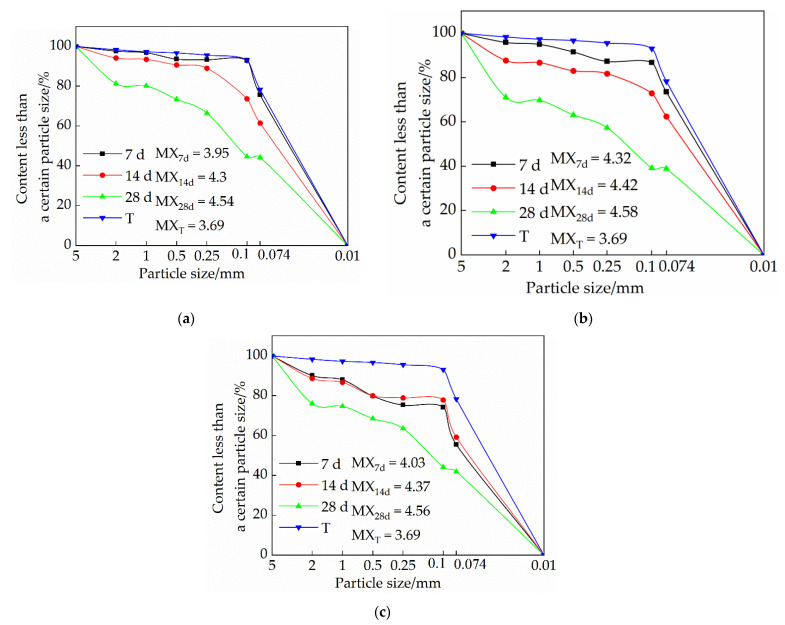
Influence of quicklime on sample particle size distribution: (**a**) Particle size distribution of samples in group S1; (**b**) Particle size distribution of samples in group S2; (**c**) Particle size distribution of samples in group S3.

Figure 7 is a graph showing the trend of the influence of quicklime on the particle gradation of the sample. It is evident from Figure 7 that under the same dosage of quicklime, the particle size of the sample increases with the increase of curing age. The addition of an appropriate amount of quicklime can effectively increase the particle size of the sample. When the content of quicklime exceeds 15%, the particle size of the sample becomes smaller. The analysis found that the calcium carbonate generated by the reaction of quicklime in the sample can play the role of binding the soil particles and making the particle size of the sample larger. When the content of quicklime exceeds 15%, a portion of it exists in the form of free ash between the pores of soil particles. Under the action of axial load, the free ash is easily broken and forms through cracks [20], resulting in a decrease in the particle size. In addition, the reaction of a large amount of quicklime in the sample will release heat, resulting in micro-cracks inside the sample, which is not conducive to the binding of soil particles, and will also cause the particle size to decrease.

#### 3.3.2. Influence of Sodium Methyl Silicate on Particle Size Distribution of Samples

**Figure 8:**
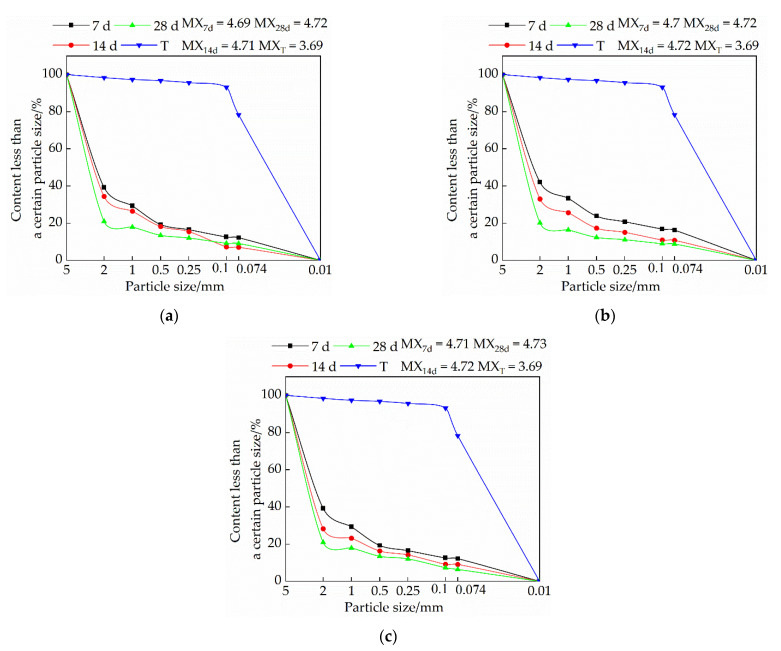
Influence of sodium methyl silicate on sample particle size distribution: (**a**) particle size distribution of samples in group J1; (**b**) particle size distribution of samples in group J2; (**c**) particle size distribution of samples in group J3.

Figure 8 is a graph showing the effect of sodium methyl silicate on the particle size distribution of the sample. It is evident from Figure 8 that the particle size of the single-doped sodium methyl silicate group is larger than that of the single-doped quicklime group; with the increase of the curing age, the particle size also becomes larger; as the content of sodium methyl silicate increases, the particle size of the sample gradually increases. An analysis of this trend reasons suggests that the polymethylsilyl (C5H12OSi) ether produced by the chemical reaction of sodium methyl silicate in the interior of the adobe can play a filling role and increase the compactness of the sample. On the other hand, polymethylsilyl (C5H12OSi) ether has a certain micro-expansion effect, which binds soil particles and increases the density.

#### 3.3.3. The Effect of Sodium Methyl Silicate and Quicklime on the Particle Size Distribution of Samples

**Figure 9:**
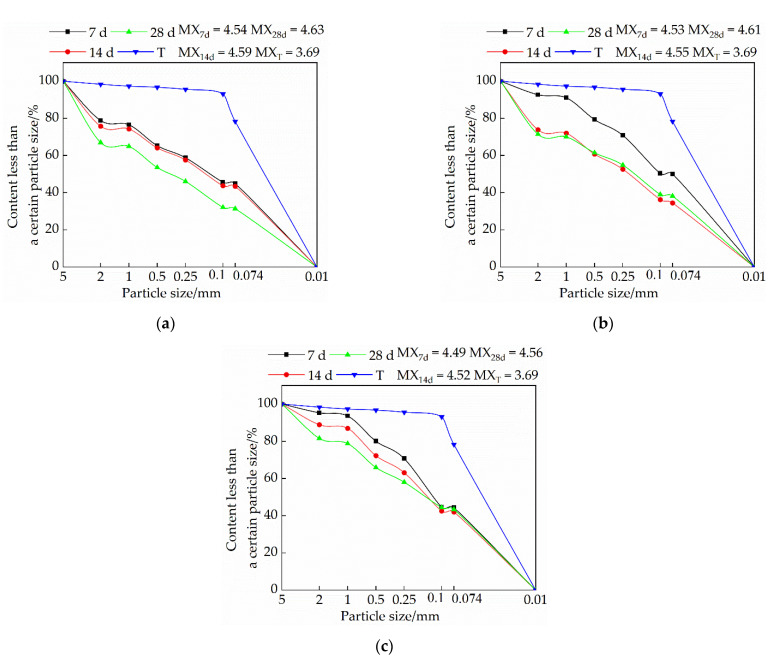
Influence of composite admixture on sample particle size distribution: (**a**) Particle size distribution of samples in group F1; (**b**) Particle size distribution of samples in group F2; (**c**) Particle size distribution of samples in group F3.

Figure 9 is a trend diagram of the effect of the composite admixture on the particle size distribution of the sample. It is evident from Figure 9 that with the increase of quicklime content, the particle size of the sample becomes increasingly smaller; under the same dosage, the particle size of the sample increases with the increase of the curing age. An analysis shows that when the amount of quicklime is high, it will consume a lot of water. In the case of rapid water loss, the amount of polymethylsilyl (C5H12OSi) ether produced by sodium methyl silicate is small, which makes the cohesion between soil particles relatively smaller; secondly, the presence of excess quicklime in the form of free ash will weaken the cohesion of soil particles. Quicklime releases heat in the process of generating CaCO_3_. When the dosage is small, the heat is negligible, and the generated CaCO_3_ will have a good effect on the soil particles between the adobe, and when the dosage is too large, the heat released will cause tiny micro-cracks inside the adobe, which will lead to the relative reduction of the cohesion between soil particles. Therefore, the higher the lime content, the smaller the particle size.

### 3.4. Effects of Mix Ratio and Curing Age on the Electrical Conductivity of Modified Raw Adobe

**Figure 10:**
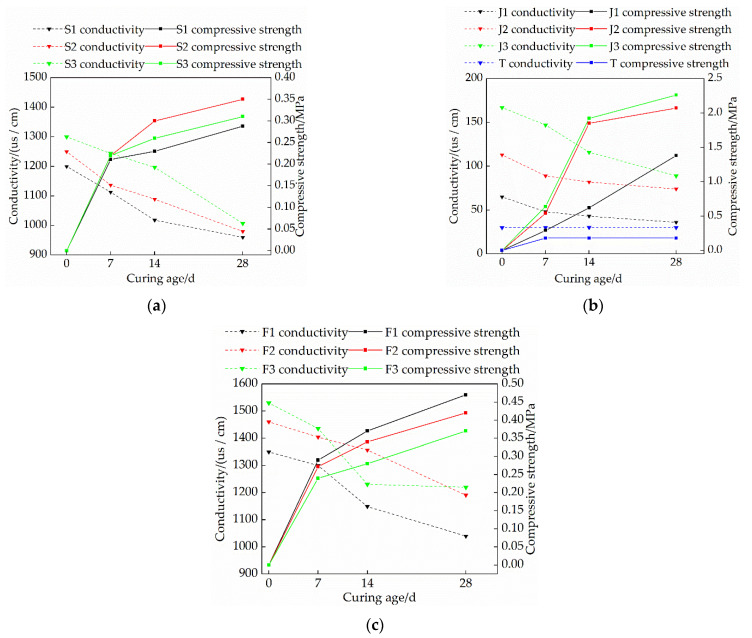
Variation of sample conductivity with curing age: (**a**) Single lime mixed group; (**b**) Mono-doped sodium methyl silicate group; (**c**) Compounding group.

The electrical conductivity of the modified raw bodies with different curing ages and mixing ratios was measured, and the results are shown in Figure 10. The conductivity of the modified soil is mainly composed of the conductivity of soil particles, CaO hydrate and carbide, sodium methyl silicate carbide, and pore water. The conductivity of the solution is positively correlated with the ion concentration in the solution [27]; therefore, in the case of the same initial moisture content, it is very important to explore the conductivity characteristics of the modified soil.

It is evident from Figure 10 that the conductivity of each group is negatively correlated with the curing age and has a high correlation [28]; at the same time, when lime or sodium methyl silicate is doped alone, the higher the content, the higher the initial conductivity, and so the compressive strength of the sample increases with the decrease of the conductivity. The reason is that under the condition of constant initial moisture content, the higher the content of lime or sodium methyl silicate, the more free ions in the sample, and the stronger the conductivity. With the increase of the curing age, the reaction in the sample continued, the free ions in the sample decreased, and the electrical conductivity decreased. As shown in Figure 10a, in the initial state, Ca(OH)_2_ was formed by lime hydration, and there were more Ca^+^ and OH^−^ ions in the solution, and the conductivity at this time was higher. With the increase in curing time, in the carbonization process, the sample further consumes free water to form CaCO_3_ crystals, which agglomerate the soil particles, hinder the flow of current to a certain extent, and reduce the conductivity of the sample.

Figure 10b is a trend graph of the conductivity of the single-doped sodium methyl silicate group as a function of curing time. In the initial state, sodium methyl silicate is fully integrated into the water, and the hydrolyzed sodium ions increase the conductivity to a certain extent. However, with the increase in the curing time, the polymethylsilyl (C5H12OSi) ether generated by the carbonation reaction of sodium methyl silicate can agglomerate soil particles and increase the compactness of the soil. The current flow is hindered to a certain extent so that the conductivity of the sample is reduced, and the strength is enhanced.

Figure 10c is a trend diagram of the effect of sodium methyl silicate and lime on the conductivity of the sample. It is evident from Figure 10c that the conductivity of the sample increases with the increase of the lime content at the same content of sodium methyl silicate; the compressive strength of the sample has a negative correlation with the conductivity. With the increase of the curing age, the electrical conductivity of the samples decreased, and the compressive strength increased. The F1 group had the lowest electrical conductivity and the highest compressive strength. In addition, for the samples of F2 and F3 groups, the electrical conductivity decreased greatly within 14 days, and the rate of decrease slowed down after 14 days. However, in the F1 group, the decreased rate of conductivity increased within 14 d~28 d. The analysis shows that when the lime content increases, the water consumption in the sample is faster, which is not enough to support the complete carbonization reaction of lime, and there will be a portion of Ca(OH)_2_, resulting in more free ions in the solution, so the conductivity rate increases. However, the lime content of the F1 group is less, and the moisture content at 14 d is also higher, which can support the further carbonization of the lime. Therefore, the F1 group can still undergo carbonization within 14 d to 28 d, and the rate of decrease in conductivity continues to increase.

### 3.5. Capillary Water Absorption Test

**Figure 11:**
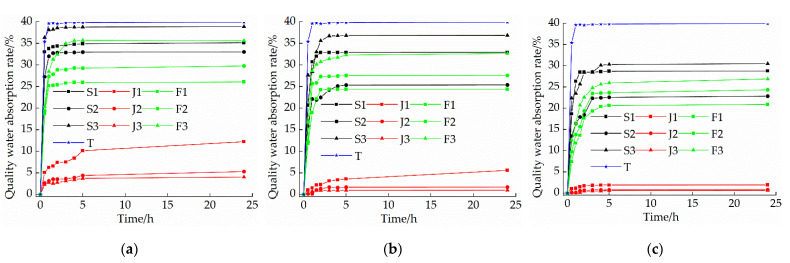
Variation of water absorption rate of sample mass with time under different curing ages: (**a**) Curing 7 d; (**b**) Curing 14 d; (**c**) Curing 28 d.

Figure 11 is the curve of the mass water absorption with the times of nine groups of samples with different ratios under different curing ages. Compared with the plain soil control group, the 24 h capillary water absorption quality of the samples in each group was significantly reduced. With the increase of the curing age, the 24 h capillary water absorption quality of each group of samples also decreased significantly. It is evident from Figure 11 that for the lime-only group, with the increase of the lime content, the capillary water absorption quality of the sample first decreased and then increased. For the sodium methyl silicate group alone, with the increase of the sodium methyl silicate content, the capillary water absorption quality of the sample decreased. For the compound blending group, with the increase of the lime content, the capillary water absorption quality of the samples increased significantly. However, the capillary water absorption quality of the samples was lower than that of the lime-mixed group. After adding sodium methyl silicate, the sample has a good waterproof effect because the silicon oxide film formed after the carbonization reaction between sodium methyl silicate and CO_2_ in the air covers the surface of the soil particles, which hinders the entry of water molecules. When the content of sodium methyl silicate increased to 1.5%, the capillary water absorption quality at the same time did not change much. The main reason for this was that when the sodium methyl silicate reached a certain amount, the generated silica film basically covered the soil particles and pores, thus after increasing the dosage, the capillary water absorption quality of the sample changed little [29]. For the compound blending group, with the increase of the lime content, the degree of expansion for the sample becomes too great, which leads to the increase of cracks in the sample and the increase in the capillary water absorption quality. In addition, too much lime content will also compete with sodium methyl silicate for CO_2_ and water in the air, resulting in a reduction in the number of silicon oxide films generated, thereby reducing the waterproof effect.

### 3.6. Micro Structure

In order to observe the evolution of the internal microstructure of the samples before and after carbonization, and the modification effect of different ratios on the raw soil, scanning electron microscope analysis was carried out on the modified raw soil samples mixed with lime, sodium methyl silicate alone, and lime and sodium methyl silicate. The results are shown in Figure 10.

**Figure 12:**
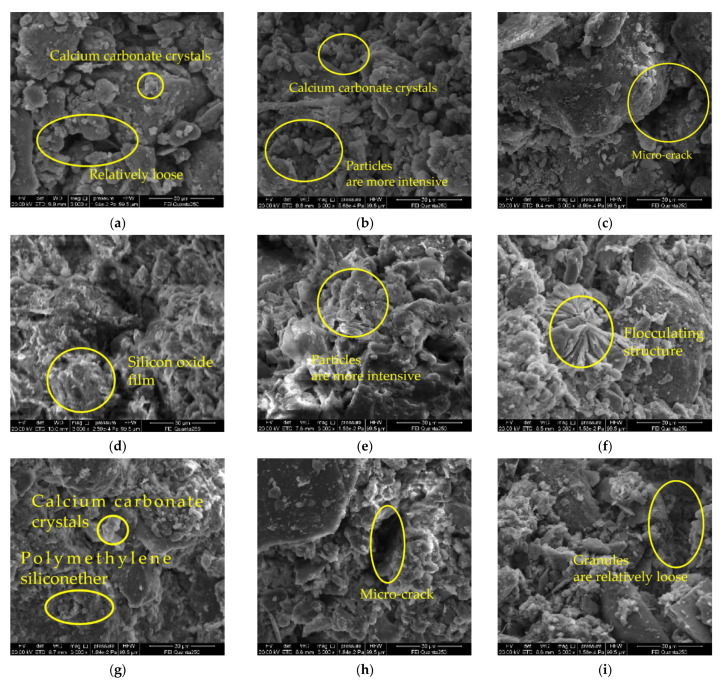
SEM pictures of different ratios of modified raw soil curing for 28 days: (**a**) 7% lime alone; (**b**) 15% lime alone; (**c**) 23% lime alone; (**d**) 0.5% mono-doped sodium methyl silicate; (**e**) 1.5% mono-doped sodium methyl silicate; (**f**) 3% mono-doped sodium methyl silicate; (**g**) 1.5% sodium methyl silicate +7% lime; (**h**) 1.5% sodium methyl silicate +15% lime; (**i**) 1.5% sodium methyl silicate +23% lime.

Figure 12a–c are the SEM images of the lime mono-doped group 28 d. It is evident from Figure 12a–c that the overall compactness of the single lime-doped group is relatively low, with obvious micro-cracks and large pores. With the increase of lime content, the Ca(OH)_2_ generated by hydration increases gradually and is plate-shaped; when the lime content is 15%, the amount of calcium carbonate generated by carbonization is high, which can better fill the pores between soil particles, increase the soil density, and play the role of binding soil particles [30]. Secondly, the pores inside the sample decrease, which can effectively prevent the entry of water molecules and improve the water-resistance of the sample. However, when the lime content exceeds 15%, there is a portion of unreacted Ca(OH)_2_ in the sample, which weakens the connection between soil particles [24].

Figure 12d–f are the SEM images of the sodium methyl silicate mono-doped group 28 d. It is evident from Figure 12d–f that with the increase of sodium methyl silicate content, more films are formed on the surface of soil particles [31], increasing amounts of flocculent crystals are formed in the soil particles, and the pores inside the soil particles decrease, and thus the density increases. When the content of sodium methyl silicate is higher than 1.5%, it signals that sodium methyl silicate has formed a relatively complete silicon oxide film in the sample. Silica film has good water resistance, mainly because the methyl groups in it are oriented and arranged to prevent the entry of water [32]. The carbonized product polymethylsilyl (C5H12OSi) ether basically filled the pores inside the sample, thereby improving the compressive strength of the sample. It is evident that sodium methyl silicate can effectively improve the compactness of the modified raw adobe, and the generated polymethylsilyl (C5H12OSi) ether can fill the pores between the soil particles. The feasibility of using sodium methyl silicate for modifying raw adobe was further verified.

Figure 12g–i are the SEM pictures of the multi-blended group after curing for 28 days. It is evident from Figure 12g–i that with the increase of the lime content, the generated calcium carbonate does increase, but the soil particles are looser, with more micro-cracks and larger pores. After the addition of sodium methyl silicate and lime, there will still be some fine micro-cracks, and the density is not as good as that of the single-doped sodium methyl silicate. According to our analysis, with the increase of the lime content, the lime will consume the water in the adobe causing a carbonization reaction and the release of a lot of heat, and the rapid disappearance of water leads to the generation of micro-cracks [33], thereby reducing the compactness and strength of the adobe. Sodium methyl silicate also consumes water when carbonization occurs, but the polymethylsilyl (C5H12OSi) ether produced by it also plays a role in water retention to a certain extent, which can slow down the rate of water loss in the adobe, thereby reducing the appearance of micro-cracks. The carbides of sodium methyl silicate and lime will have a certain micro-expansion effect. Many of the carbides agglomerate soil particles inside the sample and expand, and the expansion degree is too high, which leads to cracking inside the sample, which affects the strength and water resistance of the sample.

## 4. Discussion

Compared with the research on the soil and cultural-relic buildings in the arid regions of the Northwest, the research on the soil and cultural-relic buildings in Henan must ensure its mechanical strength on the one hand, and improve its water resistance on the other hand. Studies by Yuan et al. [34] show that sodium methylsilicate can significantly improve the mechanical properties and impermeability of silt soil, and the mechanical properties and impermeability of the improved soil increase with the increase of curing age. From the research in this paper, it is not difficult to see that the compressive strength and water resistance of the modified adobe are improved to varying degrees compared with the unmodified adobe. This is mainly because sodium methyl silicate can be decomposed into polymethylsilyl ether when encountering weak acid, and polymethylsilyl ether has the effect of micro-expanding and the enhancing the soil compactness, thereby enhancing the strength of the adobe. A large amount of polymethylsilyl ether can form a polysiloxane film between soil particles, preventing the entry of water molecules. In this way, while improving the mechanical strength of the adobe, it can also account for its water resistance. This is very beneficial for the restoration of the cultural heritage buildings in Henan Province.

Ciancio et al. [35] believed that about 5% quicklime content could improve the mechanical properties of rammed soil. Xie et al. [36] believed that about 8% quicklime could optimally improve the strength of loess. Yang et al. [14] believed that the compressive strength of the modified raw soil samples increased slowly after the lime content exceeded 10% and found that the compressive strength of the 28 d samples was the highest at 20%, followed by 15%, and the lowest at 10%. However, the content of quicklime is closely related to the size, moisture content, and production process of the prepared samples. In this paper, a large content gradient (7%, 15%, and 23%) was set for the content of quicklime to explore a suitable range for adobe in Henan. The research shows that 15% quicklime causes the greatest degree of improvement on adobe in Henan Province.

In addition, by testing the pH value, electrical conductivity, particle size distribution, and by using an SEM scanning electron microscope on the samples, it was found that when the pH value and electrical conductivity of the modified sample decreased greatly, the chemical reaction rate inside the sample was faster, and the compressive strength of the sample was enhanced. When the compressive strength of the sample was higher, the fineness modulus of the sample was larger, and the content of the large particles was also greater. This is because CaO and sodium methyl silicate can react chemically in the sample, and the resulting expanded product of calcium carbonate crystals and polymethyl silyl ether can enhance the bonding between the soil particles, thereby increasing the compressive strength of the sample. There is a certain relationship between the pH value, electrical conductivity, and compressive strength of the sample, which provides a certain scientific basis for the determination of the adobe’s curing age.

## 5. Conclusions

(1) The compressive strength of each group of modified raw adobe increased with the increase of the curing age. The compressive strengths of the samples of the single-mixed quicklime group, the single-mixed sodium methyl silicate sample, and the composite-mixed sample for 28 days were 1.94 times, 12.6 times, and 2.61 times higher than those of the plain soil samples, respectively. In addition, in the 0–14-day interval, the intensity growth rate is faster, and in the 14–28-day interval, the intensity growth rate slows down.
(2) The changing speed of pH and conductivity can express the degree of the carbonization reaction of the quicklime and sodium methylsilicate in the sample from the side. The pH and electrical conductivity of modified green adobe decreased with the increase of the curing age, decreased with the increase of the compressive strength, and the decrease range was larger from 0 d to 14 d and was smaller from 14 d to 28 d. Among them, the pH value of the 1.5% sodium methylsilicate sample decreased by 0.95 and the conductivity decreased by 31 (us/cm) within 0–14 days. For comparison, the pH value decreased by 0.32 and the conductivity decreased by 8 (us/cm) within 14–28 days. It is evident that the carbonization reaction speed of the improved sample is faster in 0–14 d, and the carbonization reaction speed is relatively slow in 14–28 d.
(3) The particle size of each group of samples increased with the increase of the curing age. The samples of the single-doped sodium methyl silicate group were the densest, and the particles larger than 2 mm were the densest. The particle size of the samples in the single-doped CaO group first increased and then decreased with the increase of the lime content. The particle size of the samples in the single-doped sodium methyl silicate group increased with the increase of the sodium methyl silicate content. The particle size of the samples in the compound blending group decreased with the increase of the lime content.
(4) It is evident from the SEM pictures that the internal pores of the samples in the modified group are all smaller, the particle structure of the sample doped with quicklime is relatively loose, and the particle structure of the sample doped with sodium methyl silicate is the densest. However, there are still micropores in the composite samples, resulting in the improvement of the strength and water resistance as “one plus one less than two”.
(5) With the addition of quicklime and sodium methyl silicate, the water-resistance of the samples was improved. Among them, the water-resistance of the sample mixed with 1.5% sodium methyl silicate alone was the best, and the capillary water absorption was only 0.79%. However, when the content of sodium methyl silicate exceeds 1.5%, the water-resistance of modified raw adobe is improved to a lesser degree.

It may be due to the different curing conditions that the combined improvement effect of quicklime and sodium methyl silicate on the sample is “one plus one less than two”. The effect of the curing conditions on the strength and water resistance of modified raw adobe will be the topic of our future research. 

## Figures and Tables

**Table 1:** Physical properties of soil.

Soil	Liquid limit/%	Plastic limit/%	Plasticity Index	Maximum Dry Density/g/cm^3^	Optimum Moisture Content/%	Natural Moisture Content/%
Silty clay	32.72	20.28	12.46	1.73	17.1	17.97

**Table 2:** Test plan.

Trial Grouping	Test Number	Wheat Straw Content/%	Curing Age/d	Lime Content/%	Sodium Methyl Silicate Content/%
	S1	0.5		7	0
Single mixed quicklime group	S2	0.5		15	0
	S3	0.5		23	0
	J1	0.5		0	0.5
Mono-doped sodium methyl silicate group	J2	0.5	7, 14, 28	0	1.5
	J3	0.5		0	3
	F1	0.5		7	1.5
Compounding group	F2	0.5		15	1.5
	F3	0.5		23	1.5
Pure soil	T	0.5	28	0	0

## Data Availability

The data presented in this study are available on request from the corresponding author.
